# α_2A_-adrenergic blockade attenuates septic cardiomyopathy by increasing cardiac norepinephrine concentration and inhibiting cardiac endothelial activation

**DOI:** 10.1038/s41598-018-23304-7

**Published:** 2018-04-03

**Authors:** Xiaohui Yu, Yuan Wang, Duomeng Yang, Xiangxu Tang, Hongmei Li, Xiuxiu Lv, Renbin Qi, Chaofeng Hu, Daxiang Lu, Ben Lv, Huadong Wang

**Affiliations:** 1 Department of Pathophysiology, Key Laboratory of State Administration of Traditional Chinese Medicine of the People’s Republic of China, School of Medicine, Jinan University, Guangzhou, Guangdong China; 2 Xiangya Hospital, Central South University, Changsha, Hunan China

## Abstract

Cardiomyopathy is a common complication associated with increased mortality in sepsis, but lacks specific therapy. Here, using genetic and pharmacological approaches, we explored the therapeutic effect of α_2A_-adrenergic receptor (AR) blockade on septic cardiomyopathy. CLP-induced septic rats were treated with BRL44408 (α_2A_-AR antagonist), prazosin (α_1_-AR antagonist) and/or reserpine. CLP-induced cardiomyopathy, indicated by reduced dP/dt and increased cardiac troponin I phosphorylation, was attenuated by BRL44408, this was associated with reduced cardiac TNF-α and endothelial VCAM-1 expression, cardiomyocyte apoptosis and related signal molecule phosphorylation. BRL44408 increased cardiac norepinephrine (NE) concentration in CLP rats. Pretreatment with reserpine that exhausts cardiac NE without affecting the circulating NE concentration or with prazosin partially abolished the cardioprotection of BRL44408 and reversed its inhibitory effects on myocardial TNF-α, apoptosis and related signal molecule phosphorylation, but not on VCAM-1 expression in septic rats. These effects of BRL44408 were confirmed by α_2A_-AR gene deletion in septic mice. Furthermore, α_2_-AR agonist not only enhanced LPS-induced TNF-α and VCAM-1 expression in cardiac endothelial cells that express α_2A_-AR, but also enhanced LPS-induced cardiac dysfunction in isolated rat hearts. Our data indicate that α_2A_-AR blockade attenuates septic cardiomyopathy by promoting cardiac NE release that activates myocardial α_1_-AR and suppressing cardiac endothelial activation.

## Introduction

Septic cardiomyopathy is a common complication associated with increased mortality in septic patients^1^. Numerous studies have demonstrated that multiple factors contribute to septic cardiomyopathy, including myocardial tumor necrosis factor-α (TNF-α), vascular cell adhesion molecule-1 (VCAM-1) and cardiomyocyte apoptosis^2–9^. During sepsis, pathogen-associated molecular patterns, such as lipopolysaccharide (LPS), stimulate Toll-like receptors and induce myocardial inflammatory cytokine production and apoptosis via activating nuclear factor (NF)-κB and mitogen-activated protein kinases (MAPK), the extracellular signal-regulated kinase (ERK1/2), p38 and c-Jun NH2-terminal kinase (JNK)^3,4^. For example, it has been demonstrated that myocardial TNF-α expression significantly increases in animal models of LPS or caecal ligation and puncture (CLP)-induced sepsis, and inhibition of p38 or TNF reduces sepsis-induced myocardial dysfunction^4–6^. LPS and CLP challenge also upregulate myocardial VCAM-1 expression in mice. Blockade of VCAM-1 ameliorates LPS-induced myocardial dysfunction^7,8^. Similarly, LPS activates cardiomyocyte apoptosis and induces myocardial dysfunction, which are completely prevented by treatment with broad spectrum caspase inhibitor^9^. However, the pathogenesis of septic cardiomyopathy is complex. Although it has been studied for more than 50 years, the exact underlying mechanisms remain elusive and no specific, effective therapy currently exists. Thus, additional studies are necessary to identify therapeutic targets for myocardial dysfunction induced by sepsis.

Recently, a specific antagonist for the α_2A_-adrenergic receptor (α_2A_-AR), 2-[(4,5-dihydro-1H-imidazol-2-yl) methyl]-2,3-dihydro-1-methyl-1H-isoindole maleate (BRL44408, BRL), was found to reduce proinflammatory cytokine production and mortality in septic rats^10^. In addition, we demonstrated that treatment with yohimbine, an α_2_-AR antagonist, promoted cardiac norepinephrine (NE) release and attenuated lipopolysaccharide (LPS)-induced cardiac dysfunction probably through blocking cardiac presynaptic α_2_-AR^11^. Despite these investigations, the causal contribution of α_2A_-AR to septic cardiomyopathy has never been examined.

Therefore, we hypothesize that α_2A_-AR activation is involved in septic cardiomyopathy and that α_2A_-AR blockade attenuates sepsis-induced cardiomyopathy by promoting cardiac NE release and suppressing myocardial inflammation and apoptosis. Here, using gene knockout technique and pharmacological antagonist, we first investigated the effect of α_2A_-AR blockade on cardiac NE release, myocardial inflammation, apoptosis and myocardial dysfunction during sepsis; Secondly, we used reserpine (RSP) as a tool to exhaust cardiac NE concentration and prazosin (PRAZ) to block α_1_-AR to test whether increased concentration of cardiac NE and subsequent α_1_-AR activation mediated cardioprotection of BRL44408 in CLP rats; Lastly, we observed that the effects of α_2_-AR agonist, BHT933 (BHT), on cardiac endothelial inflammation and cardiac function in cultured cardiac endothelial cells and isolated rat hearts treated with LPS.

## Results

### BRL therapy improves survival and left ventricular function in septic rats

**Figure 1:**
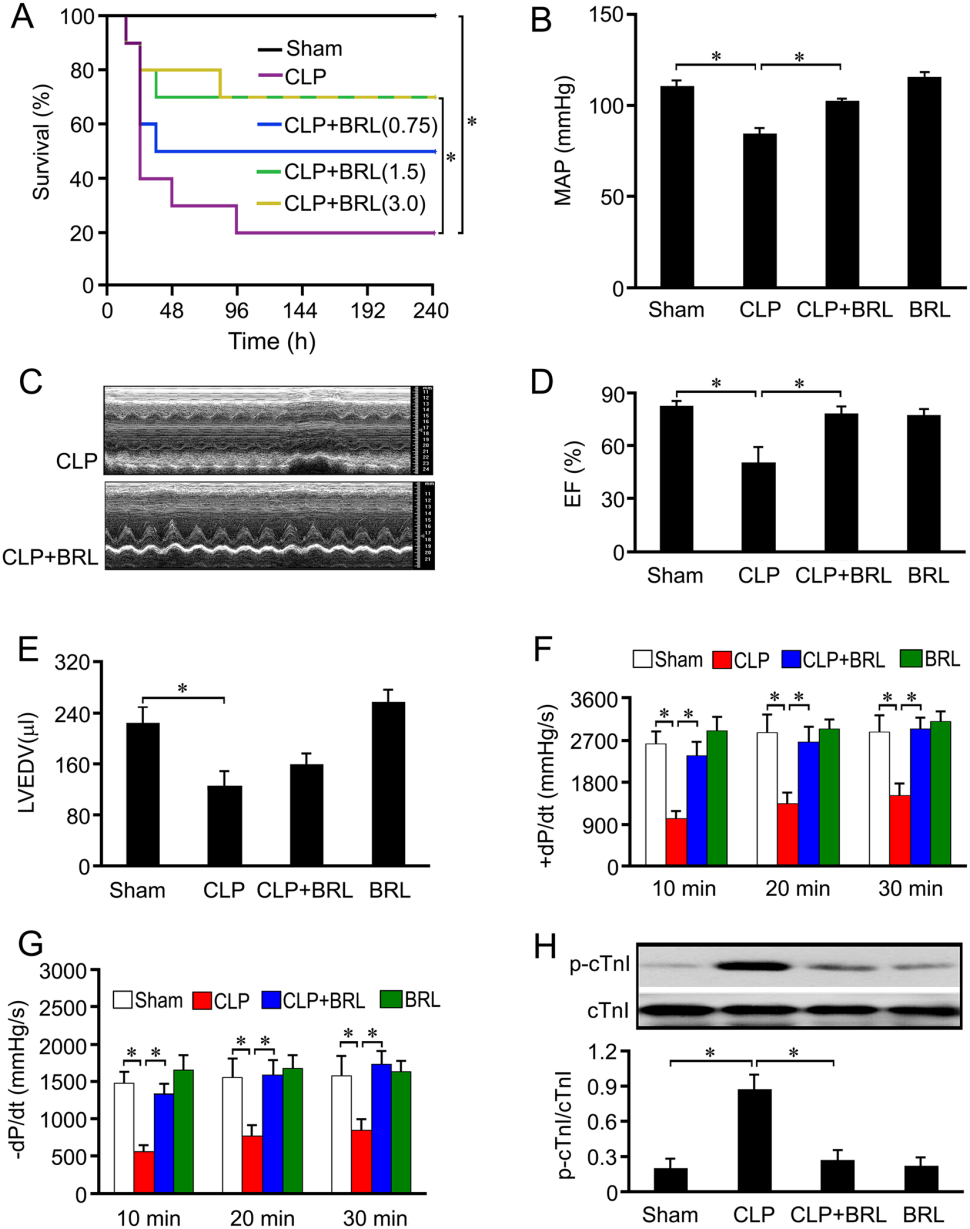
BRL44408 (BRL) therapy improves survival and reverses cardiac dysfunction and hypotension in septic rats. The rats were randomized into sham, cecal ligation and puncture (CLP), CLP+BRL and sham+BRL (BRL) groups. The rats were subjected to CLP or sham surgery and were treated intraperitoneally with either BRL (i.p.) or normal saline 4 h later. (**A**): A Kaplan-Meier plot indicating that treatment with BRL (1.5 or 3.0 mg/kg) significantly improves the survival of septic rats (*n* = 10 for each group). (**B**): The mean arterial pressure (MAP) was measured 20 h after CLP or sham surgery using the tail-cuff method, and BRL (1.5 mg/kg) elevated the MAP in septic rats. (**C**,**D**) and (**E**): Echocardiography evaluation indicated that BRL (1.5 mg/kg) treatment increased left ventricular ejection fraction (EF), but not left ventricular end-diastolic volume (LVEDV) in septic rats. (**F**) and (**G**): The hearts were obtained 20 h after CLP or sham surgery and left ventricular function was assessed in a Langendorff apparatus. The maximum rates of the rise (+dP/dt) and fall (−dP/dt) of the left ventricular pressure 30 min after perfusion are shown. BRL (1.5 mg/kg) increased +dP/dt and −dP/dt in CLP rats. (**H**): Left ventricle samples were obtained for the Western blot analysis 20 h after CLP or sham surgery, and BRL (1.5 mg/kg) inhibited left ventricular cardiac troponin I (cTnI,) phosphorylation (Ser23/24) in septic rats. *n* = 7 for the sham and the BRL groups, *n* = 10 for the CLP and the CLP+BRL groups. Statistical significance is shown as *P < 0.05.

To block the α_2A_-AR, we first used a selective α_2A_-AR antagonist, BRL. As shown in Fig. 1A, the intraperitoneal administration of BRL (1.5 mg/kg or 3.0 mg/kg) 4 h post-CLP significantly improved survival in septic rats (*P* < 0.05). At 20 h post-CLP, the septic rats showed a marked drop in mean arterial pressure (MAP), which was attenuated by 1.5 mg/kg BRL treatment (Fig. 1B). Echocardiography revealed that left ventricular (LV) EF (Fig. 1D) and LVEDV (Fig. 1E) markedly decreased at 20 h after CLP induction in septic rats, which were attenuated by BRL treatment except LVEDV. The LV ± dP/dt values (Fig. 1F,G) of the hearts from septic rats 20 h post-CLP decreased significantly compared with the sham-operated rats, which were corrected by 1.5 mg/kg BRL. In particular, cardiac contractile depression is associated with the increased phosphorylation of cTnI in sepsis^12^ and BRL (1.5 mg/kg) significantly suppressed LV cTnI phosphorylation in septic rats (Fig. 1H). Moreover, BRL (1.5 mg/kg) did not significantly affect the above parameter in the sham-operated rats at 20 h after sham surgery. These data indicate that the pharmacological inhibition of α_2A_-AR can attenuate septic myocardial dysfunction.

### BRL reduces myocardial TNF-α production and apoptosis as well as IκBα, JNK and p38 phosphorylation in septic rats

**Figure 2:**
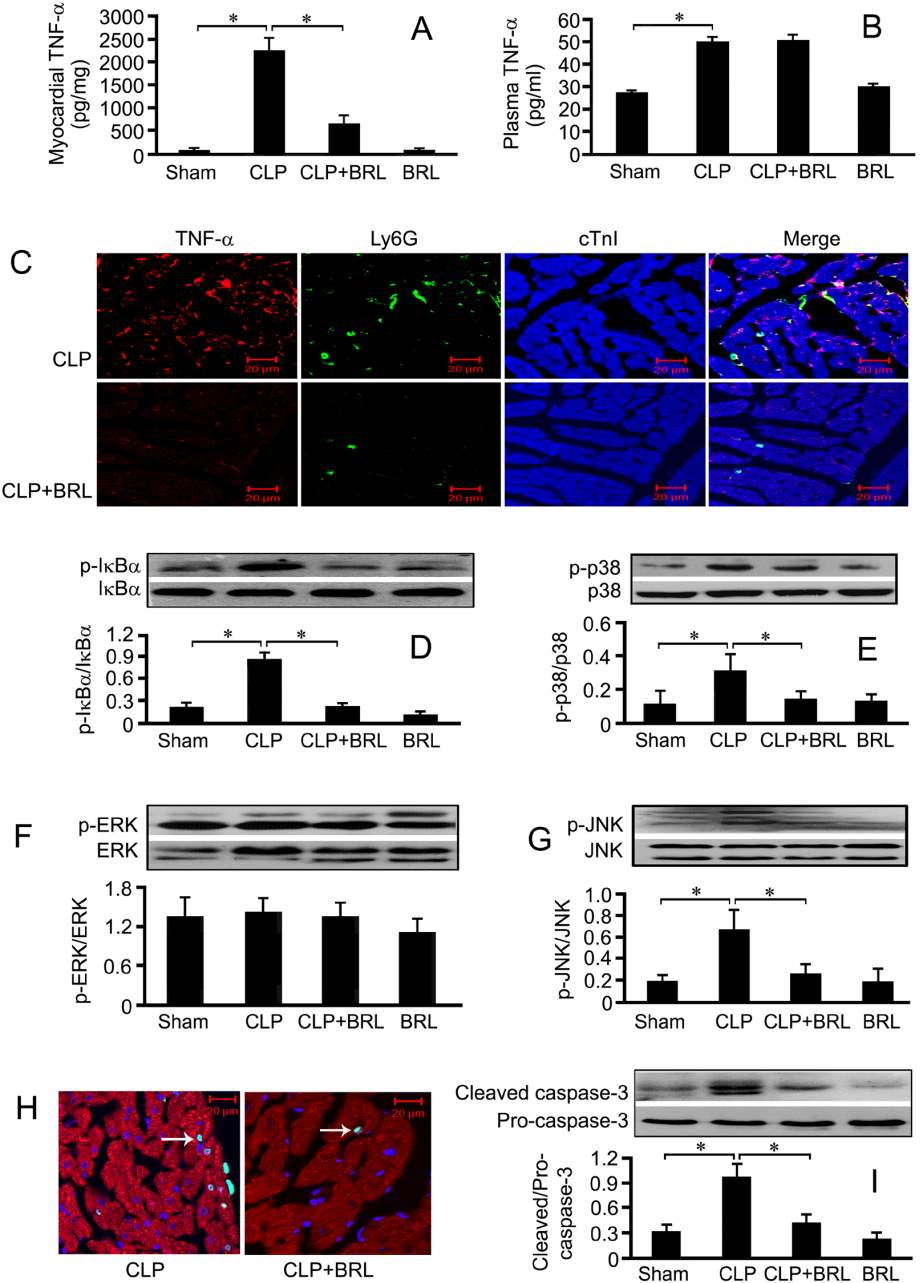
BRL44408 (BRL) therapy suppresses myocardial tumor necrosis factor (TNF) - α production, neutrophil accumulation as well as inhibitors of κBα (IκBα), p38 and c-Jun N-terminal kinases (JNK) phosphorylation and inhibits cardiomyocyte apoptosis in CLP-induced septic rats. The rats were randomized into sham, CLP, CLP+BRL and sham+BRL (BRL) groups. Then, the rats were subjected to CLP or sham surgery and were treated with BRL (1.5 mg/kg, i.p.) or normal saline 4 h later. Left ventricle samples and blood were obtained for analysis 20 h after CLP or sham surgery. Myocardial (**A**) and plasma (**B**) TNF-α concentrations were detected by ELISA. (**C**): Representative confocal images of cardiac sections, the sections were stained with antibodies against TNF-α (red), Ly6G (a neutrophil marker, green) and cardiac troponin I (cTnI, blue). Phosphorylation of IκBα (**D**), p38 (**E**), extracellular signal-regulated kinase 1/2 (ERK1/2, **F**) and JNK (**G**) were detected by Western blot analysis. (**H**): Representative confocal images of cTnI (red), DAPI (blue) and terminal deoxynucleotidyl transferase dUTP nick-end labeling (TUNEL) reagents (green)-stained cardiac sections are shown from the CLP and the CLP+BRL groups, respectively. Arrows indicate TUNEL-positive nuclei. (**I**): Western blot quantification of myocardial cleaved caspase-3. *n* = 8 for each group. Statistical significance is shown as *P < 0.05.

MAPK and NF-κB signal pathways-mediated myocardial TNF-α production and apoptosis contribute to the sepsis-induced myocardial dysfunction. As shown in Fig. 2A,B, cardiac and plasma TNF-α concentrations increased in the septic rats at 20 h after CLP. BRL (1.5 mg/kg) significantly decreased cardiac, but not plasma TNF-α concentrations in CLP rats. Immunofluorescence staining showed that BRL inhibited CLP-induced myocardial TNF-α expression (Fig. 2C). Moreover, septic rats displayed markedly elevated phosphorylation levels of IκBα (Fig. 2D), p38 (Fig. 2E) and JNK (Fig. 2G) in the left ventricular myocardium at 20 h after CLP surgery, which were decreased by BRL treatment. Phosphorylation of ERK had no obvious changes in the CLP rats treated with and without BRL (Fig. 2F). TUNEL assay showed that more apoptotic cardiomyocytes were observed at 20 h after CLP surgery in the vehicle-treated rats than BRL-treated CLP rats (Fig. 2H). Similarly, CLP caused a significant increase in myocardial cleaved caspase-3 concentration, which was inhibited by BRL therapy (Fig. 2I).

### BRL inhibits sepsis-induced cardiac neutrophil infiltration and endothelial VCAM-1 expression

**Figure 3:**
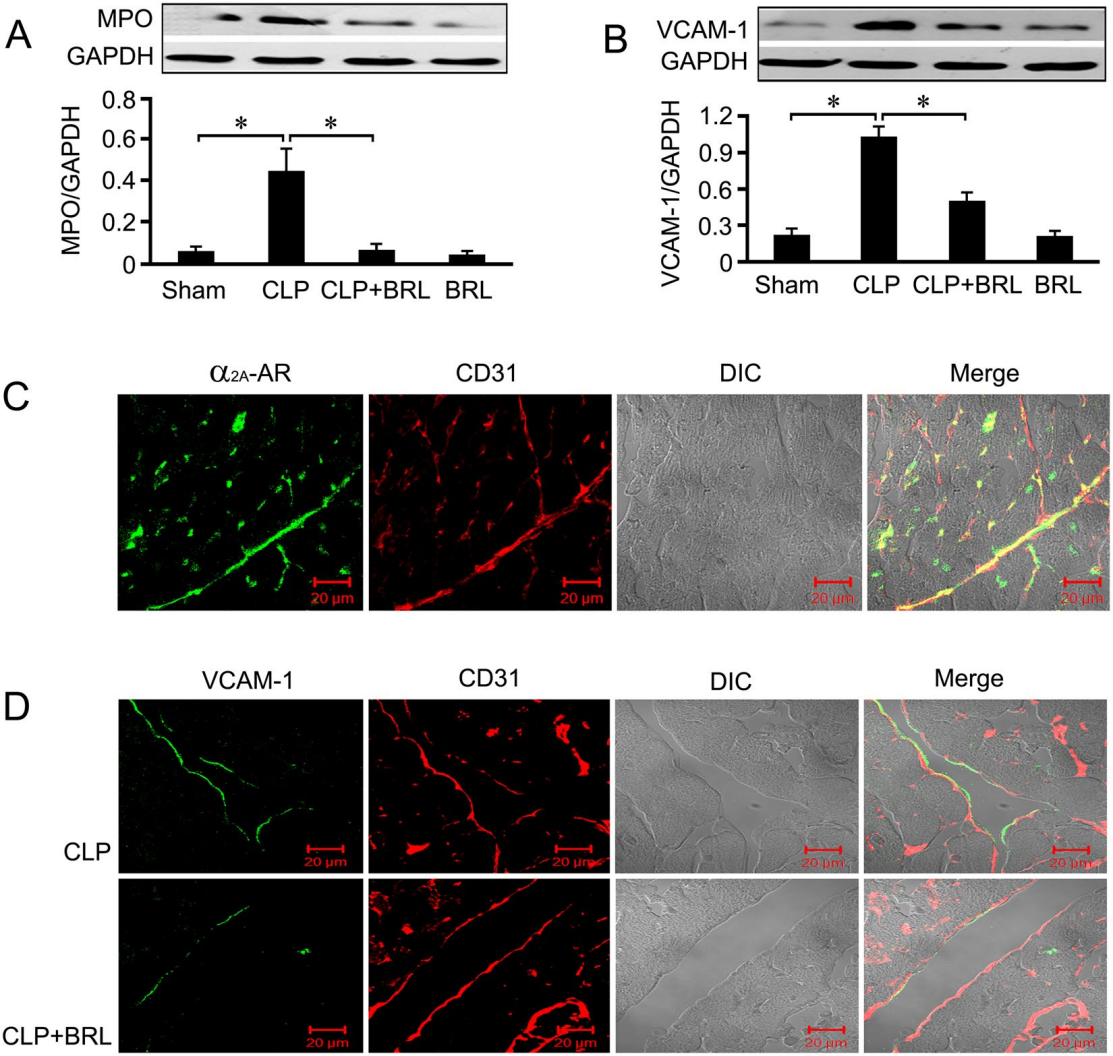
BRL44408 (BRL) treatment reduces the contents of cardiac myeloperoxidase (MPO) and endothelial vascular cell adhesion molecule (VCAM)-1 in CLP-induced septic rats. The rats were administered either normal saline or BRL (1.5 mg/kg) 4 h after CLP or sham surgery. Whole-tissue lysates were prepared from the left ventricle at 20 h after CLP or sham surgery, and MPO (**A**) and VCAM-1 (**B**) concentrations within the myocardium were measured by Western blot analysis. GAPDH was used as loading control. *n* = 8 for each group. Statistical significance is shown as *P < 0.01. (**C**): Immunofluorescence staining of α_2A_-AR (green) and CD31 (a positive marker for endothelial cells, red) in the left ventricle sections from the sham group at 6 h after sham surgery. DIC indicates differential interference contrast. Scale bar = 20 μm. (**D**): Immunofluorescence staining of VCAM-1 (green) and CD31 (red) in the left ventricle sections from septic rats at 6 h after the CLP challenge. DIC indicates differential interference contrast. Scale bar = 20 μm.

Neutrophil infiltration was examined by combining immunofluorescence labeling for Ly6G and cTnI. As shown in Fig. 2C, BRL decreased myocardial neutrophil infiltration in septic rats at 20 h after CLP, which was further confirmed by the cardiac myeloperoxidase (MPO) content (Fig. 3A). Furthermore, cardiac VCAM-1 expression markedly increased in CLP rats, which was suppressed significantly by BRL (Fig. 3B). As demonstrated in Fig. 3C, α_2A_-AR is present in the cardiac endothelial cells. VCAM-1-positive endothelial cells increased in the left ventricle sections of septic rats 6 h after CLP and decreased in septic rats treated with BRL (Fig. 3D).

### BRL increases left ventricular norepinephrine concentration in CLP rats

**Figure 4:**
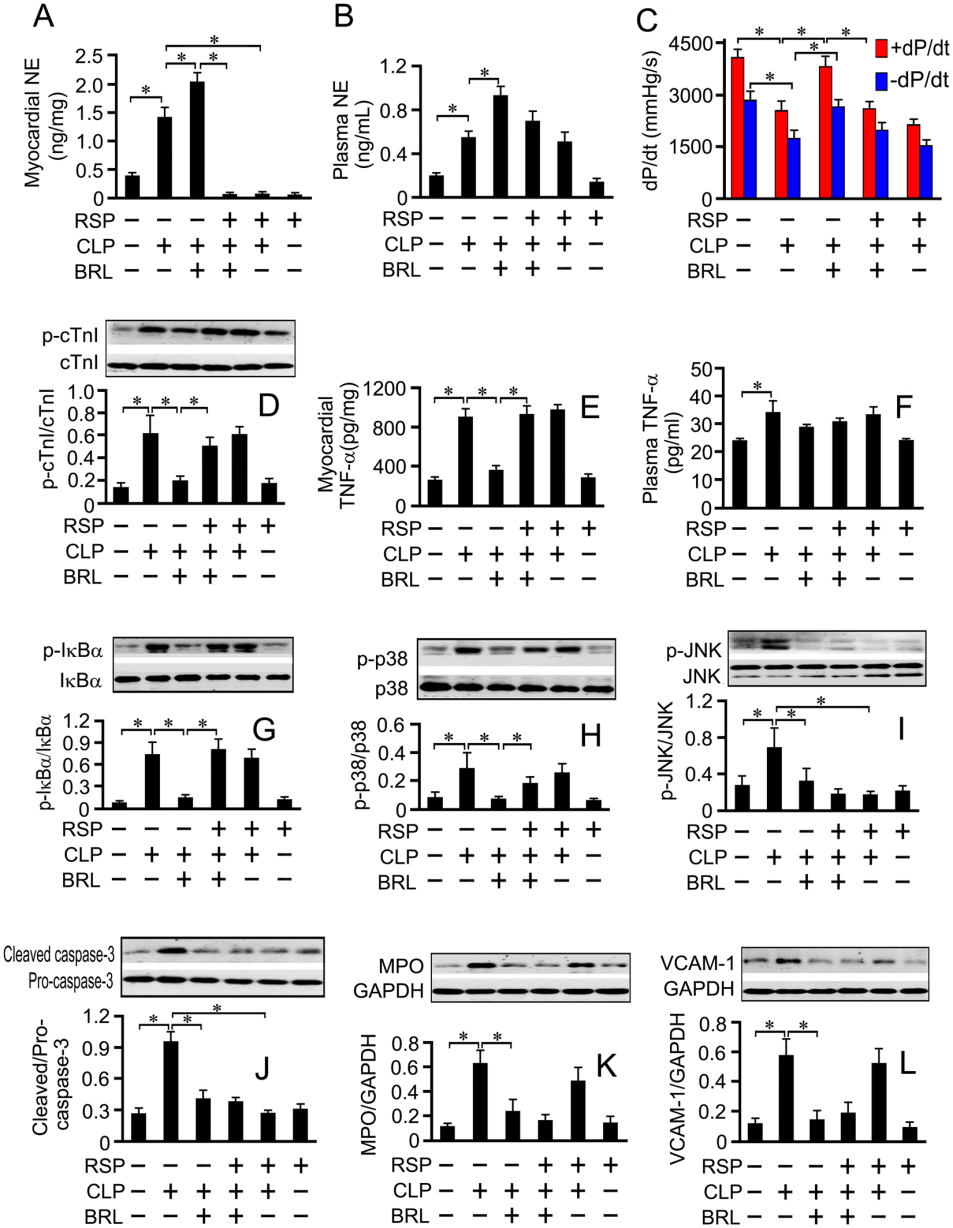
Reserpine (RSP) pretreatment exhausts cardiac norepinephrine (NE) and partially reverses the protective effect of BRL44408 (BRL) on the heart in CLP-treated rats. Rats first received subcutaneous injections of RSP (4.5 mg/kg) or normal saline once a day for 2 consecutive days and were then exposed to CLP or sham surgery on the 4th day after the last RSP administration. BRL (1.5 mg/kg) or normal saline was injected intraperitoneally 4 h after CLP or sham surgery. Cardiac (**A**) and plasma (**B**) NE concentrations were detected by ELISA at 6 h after CLP or sham surgery. At 20 h after CLP or sham surgery, (**C**) left ventricular function was assessed in a Langendorff apparatus, the maximum rates of the rise (+dP/dt) and fall (−dP/dt) of the left ventricular pressure at 30 min after perfusion are shown; (**D)** The left ventricular phosphorylated cTnI (Ser23/24) levels were detected by Western blot analysis; Left ventricular (**E**) and plasma (**F**) tumor necrosis factor (TNF)-α concentrations were detected by ELISA; Left ventricular myocardial IκBα (**G**), p38 (**H**) and JNK (**I**) phosphorylation, caspase-3 activation (**J**) as well as MPO (**K**) and VCAM-1 (**L**) levels were detected by Western blot analysis. *n* = 7 for the sham or RSP group; *n* = 10 for the CLP, CLP+BRL, RSP+CLP+BRL and RSP+CLP groups. Statistical significance is shown as *P < 0.05.

Since α_2A_-AR is responsible for presynaptic feedback inhibition of NE release, we further investigated the changes in NE concentrations in the left ventricle and plasma. As shown in Fig. 4A,B, both plasma and left ventricle NE concentrations were significantly increased at 6 h after CLP and were further elevated by BRL therapy.

### Cardiac NE depletion by reserpine partially abolishes the cardioprotection of BRL in septic rats

To test whether increased cardiac NE mediates the cardioprotection of BRL treatment, we used reserpine (RSP) as a tool to exhaust cardiac NE in CLP rats. It was reported that there was no NE recovery in heart from the 4^th^ day to 10^th^ day after subcutaneous injection of RSP in rats^13^. In the present study, the rats received subcutaneous injections of RSP (4.5 mg/kg) once a day for 2 consecutive days and were exposed to CLP or sham surgery on the 4th day after the last RSP administration. BRL (1.5 mg/kg) or normal saline was injected 4 hours after CLP or sham surgery. At 6 h after CLP, BRL increased plasma and cardiac NE concentrations in septic rats, but left ventricular NE was depleted by RSP in septic rats treated with BRL. No significant difference was observed in plasma NE concentrations between the RSP+CLP+BRL and the CLP+BRL groups (Fig. 4A,B).

In particular, RSP partially abolished the cardioprotection of BRL in septic rats, as evidenced by reduced LV+dP/dt and increased cTnI phosphorylation (Fig. 4C,D). RSP did not affect the plasma TNF-α concentration in septic rats treated with or without BRL (Fig. 4F). However, RSP reversed the inhibitory effects of BRL on myocardial TNF-α production, without affecting the myocardial TNF-α concentration in septic rats (Fig. 4E). Similar results were obtained for myocardial IκBα and p38 phosphorylation (Fig. 4G,H). In contrast, RSP did not abrogate the inhibitory effects of BRL on myocardial JNK phosphorylation and caspase-3 activation or on MPO and VCAM-1 concentrations in septic rats, but inhibited myocardial JNK phosphorylation and caspase-3 activation in CLP rats (Fig. 4I–L).

### α_1_-AR blockade also partially abrogates the cardioprotection of BRL in CLP rats

**Figure 5:**
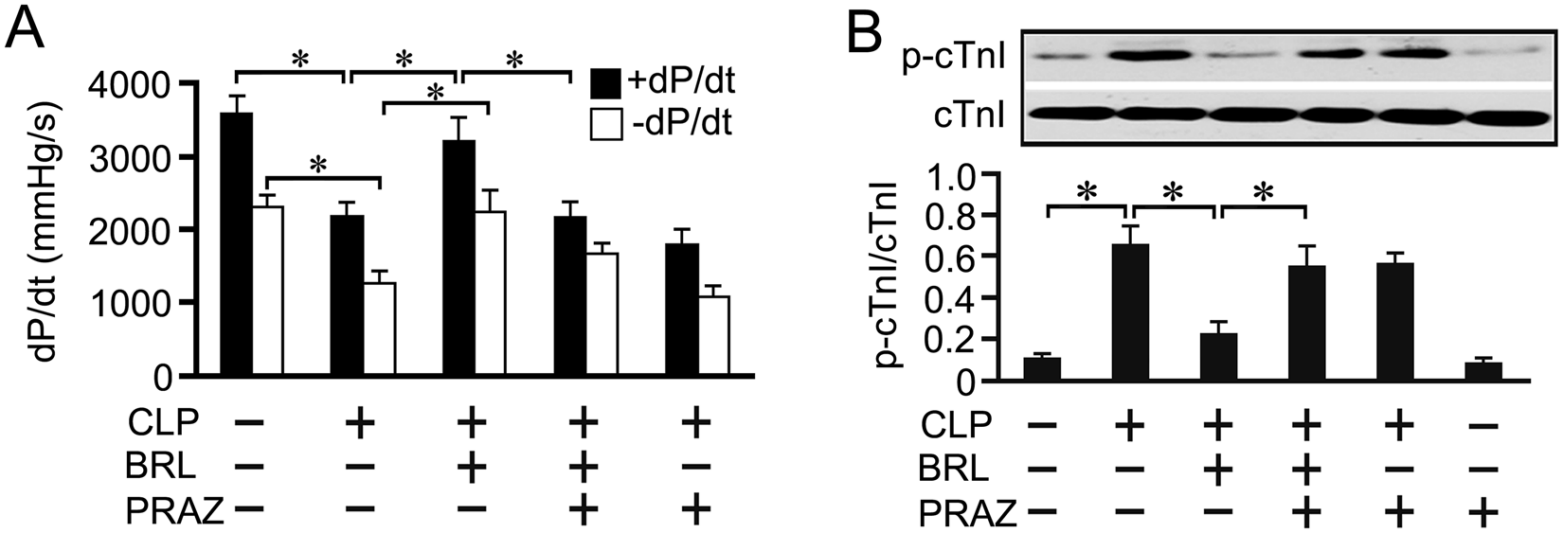
Prazosin (PRAZ, a selective α_1_-AR antagonist) administration partially abrogates the inhibitory effect of BRL44408 (BRL) on the cardiac dysfunction in CLP-treated rats. Rats received intraperitoneal injections with normal saline, BRL (1.5 mg/kg) or/and PRAZ (1.0 mg/kg) at 4 h after CLP or sham surgery. Left ventricular function was assessed in a Langendorff apparatus and whole-tissue lysates from the left ventricle were prepared at 20 h after CLP or sham surgery. (**A**): The maximum rates of rise (+dP/dt) and fall (−dP/dt) of the left ventricular pressure at 30 min after perfusion are shown. (**B**): Phosphorylated cTnI (Ser23/24) concentrations were detected by Western blot assay. *n* = 7 for the sham or PRAZ group; *n* = 10 for the CLP, CLP+BRL, CLP+BRL+PRAZ and CLP+PRAZ groups. Statistical significance is shown as *P < 0.05.

**Figure 6:**
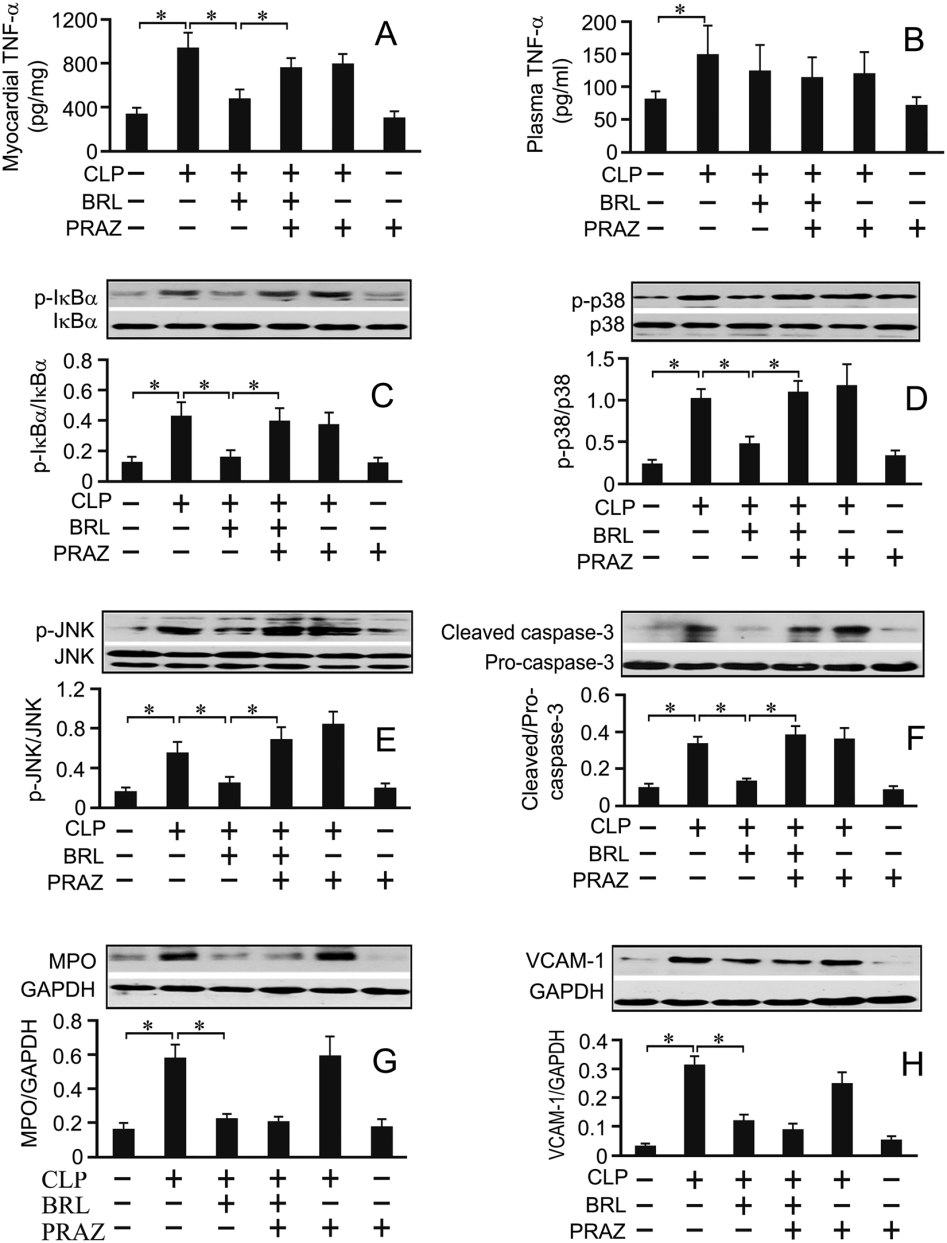
The effects of prazosin (PRAZ) or/and BRL44408 (BRL) on left ventricular and plasma TNF-α, left ventricular IκBα, p38 and JNK phosphorylation, caspase-3 activation as well as left ventricular myeloperoxidase (MPO) and VCAM-1 contents in CLP-treated rats. The rats received intraperitoneal injection with normal saline, BRL (1.5 mg/kg) or/and PRAZ (1.0 mg/kg) at 4 h after CLP or sham surgery. Plasma and whole-tissue lysates from the left ventricle were prepared at 20 h after CLP or sham surgery. Left ventricular (**A**) and plasma (**B**) TNF-α concentrations were detected by ELISA. Left ventricular IκBα (**C**), p38 (**D**) and JNK (**E**) phosphorylation, caspase-3 activation (**F**) as well as MPO (**G**) and VCAM-1 (**H**) were detected by Western blot assay. *n* = 7 for the sham or PRAZ group; *n* = 10 for the CLP, CLP+BRL, CLP+BRL+PRAZ and CLP+PRAZ groups. Statistical significance is shown as *P < 0.05.

To investigate which receptor is involved in cardioprotection with increased myocardial NE release by BRL during sepsis, prazosin (PRAZ), an α_1_-AR antagonist, was administered just before BRL. As shown in Fig. 5A,B, PRAZ partially abolished the cardioprotection of BRL in septic rats, as evidenced by reduced LV+dP/dt and increased cTnI phosphorylation at 20 h after CLP. PRAZ did not alter the plasma TNF-α concentration in septic rats treated with or without BRL (Fig. 6B). Although PRAZ did not affect myocardial TNF-α production, caspase-3 activation and IκBα, p38 and JNK phosphorylation in septic rats, it eliminated the suppressive effects of BRL on myocardial TNF-α production (Fig. 6A), caspase-3 activation (Fig. 6F) and on IκBα, p38 and JNK phosphorylation (Fig. 6C–E) in septic rats. However, PRAZ did not reverse the inhibitory effects of BRL on cardiac MPO and VCAM-1 expression in CLP rats (Fig. 6G,H).

### Genetic deletion of α_2A_-AR mimics the cardioprotection of BRL in septic mice

**Figure 7:**
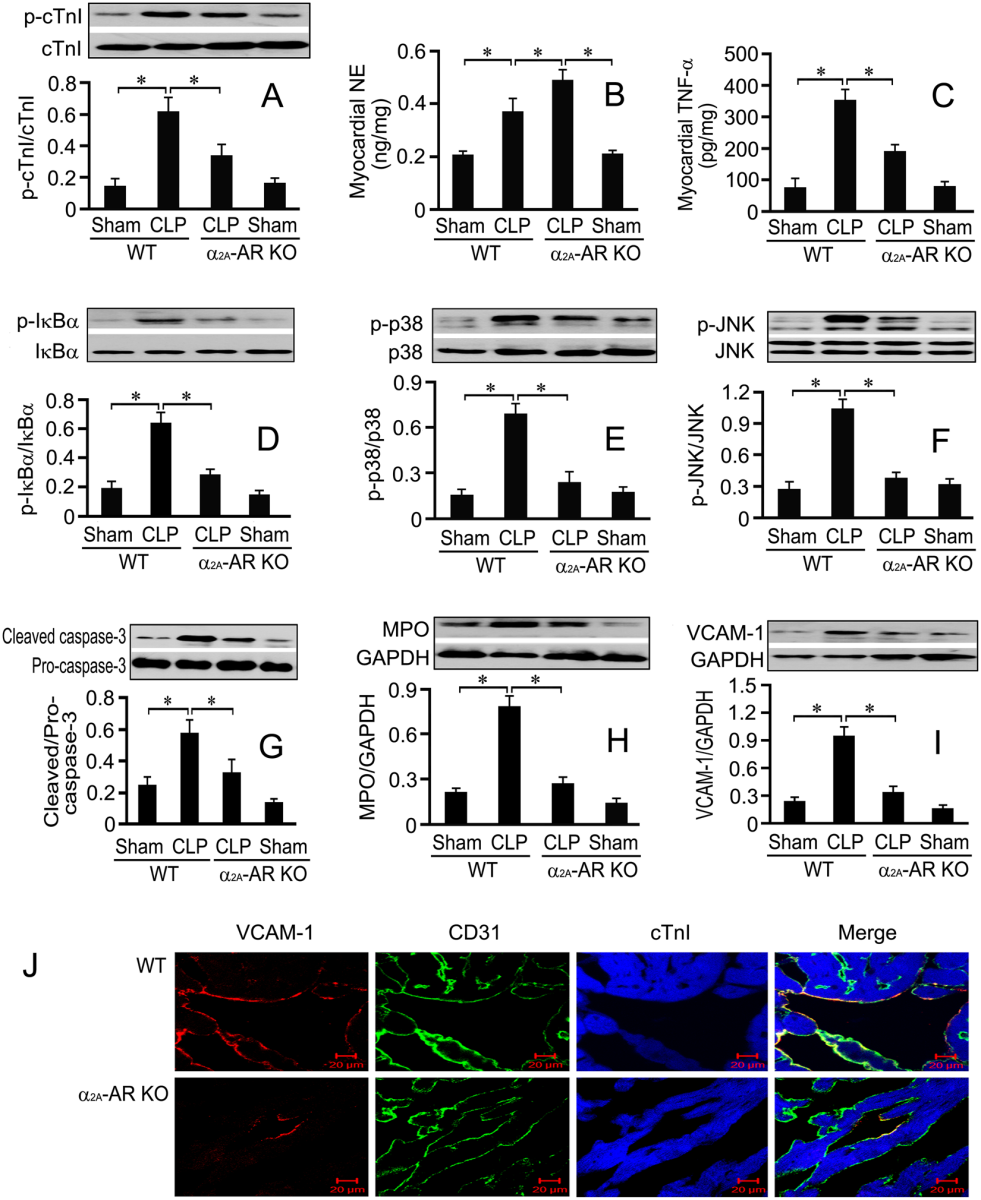
α_2A_-AR knockout (KO) mice show significantly increased cardiac norepinephrine (NE) level and reduced myocardial inflammation, apoptosis and cardiac dysfunction induced by CLP. α_2A_-AR KO mice and their wild-type (WT) counterparts received CLP or sham surgery, sixteen hours later, left ventricle samples were prepared. (**A**): Phosphorylated cTnI (Ser23/24) concentrations were detected by Western blot assay. Cardiac NE (**B**) and TNF-α (**C**) concentrations were detected by ELISA. Left ventricular IκBα (**D**), p38 (**E**) and JNK (**F**) phosphorylation, caspase-3 activation (**G**) as well as MPO (**H**) and VCAM-1 (**I**) concentrations were detected by Western blot. *n* = 9 for each group. Statistical significance is shown as *P < 0.05. (**J**): Representative confocal images of left ventricular sections from α_2A_-AR KO mice and their WT counterparts exposed to CLP. The left ventricular slices were stained with antibodies against VCAM (red), CD31 (a marker for endothelial cells, green) and cardiac troponin I (cTnI, blue). Scale bar = 20 μm.

As shown in Fig. 7, the genetic deletion of α_2A_-AR reduced CLP-induced LV cTnI phosphorylation in mice (Fig. 7A). WT mice subjected to CLP showed significantly increased cardiac NE concentrations, which were enhanced in α_2A_-AR KO mice (Fig. 7B). CLP enhanced the myocardial TNF-α concentration and caspase-3 activation, with simultaneously increased myocardial IκBα, p38 and JNK phosphorylation in WT mice, all of which were reduced in α_2A_-AR KO mice (Fig. 7C–G). CLP also increased cardiac VCAM-1 and MPO concentrations in WT mice, which were reduced in α_2A_-AR KO mice (Fig. 7H,I). Moreover, immunofluorescence staining showed that α_2A_-AR deficiency reduced CLP-induced cardiac endothelial VCAM-1 expression (Fig. 7J).

### α_2_-AR agonist promotes LPS-induced cardiac endothelial activation and myocardial dysfunction of isolated rat hearts

**Figure 8:**
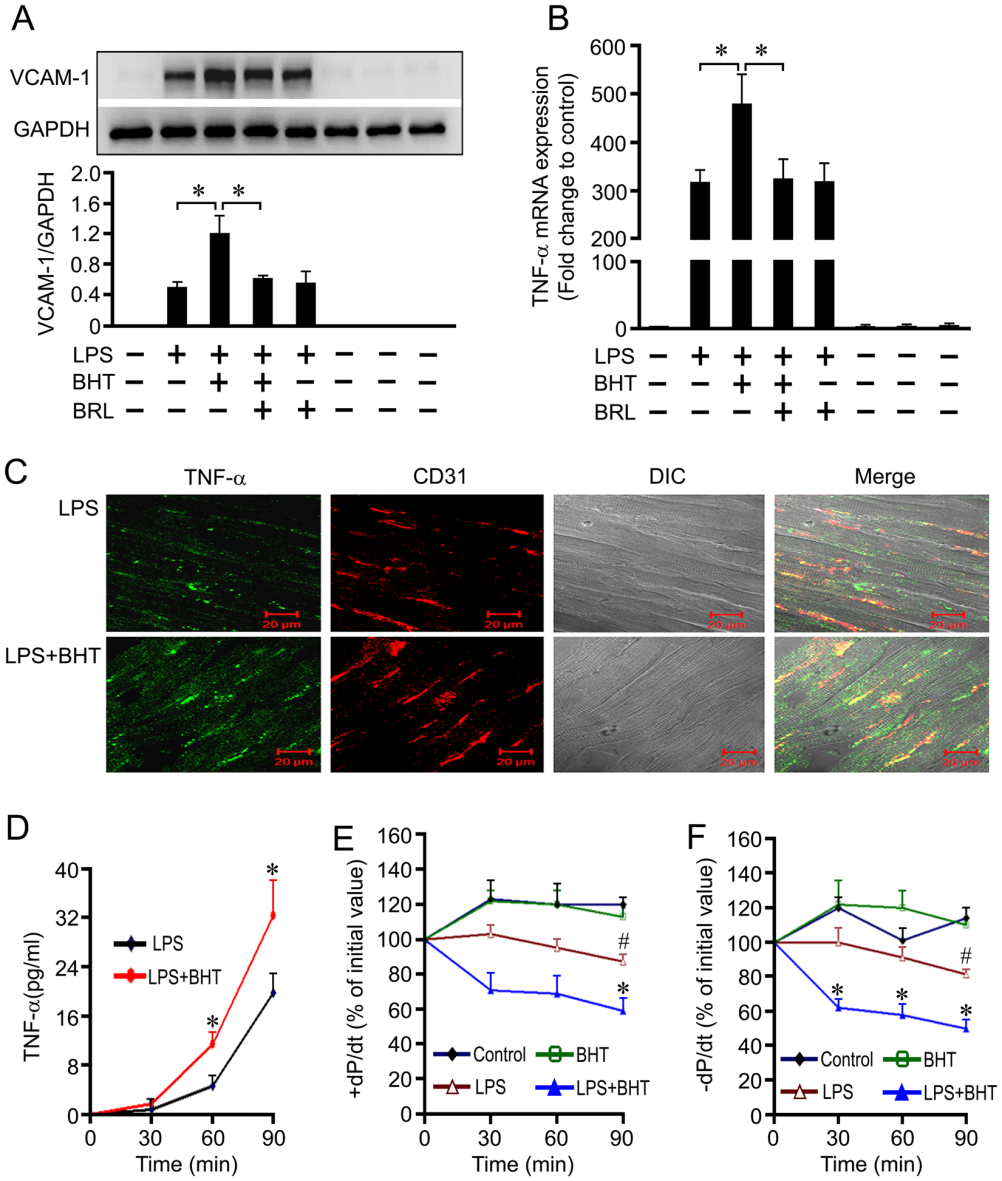
Treatment with BHT933 (BHT), a selective α_2_-AR agonist, enhances lipopolysaccharide (LPS)-induced cardiac endothelial cell activation and cardiac dysfunction. (**A**) and (**B**): Purified cardiac microvascular endothelial cells were preincubated with BRL44408 (BRL) or vehicle for 30 min and with vehicle or BHT933 (BHT) for another 30 min, and then treated with or without LPS for 12 or 2 h, the expression of VCAM-1 protein and TNF-α mRNA were determined. *n* = 4 for each group. *P < 0.05. (**C**): Immunofluorescence staining of TNF-α (green) and CD31 (red) in the left ventricular sections from isolated hearts perfused with LPS or LPS+BHT for 1 h. Scale bar = 20 μm. (**D**,**E**) and (**F**): All hearts were perfused in a recirculating mode (total volume, 50 mL) via the aorta with oxygenated (95% O_2_: 5% CO_2_) Krebs-Henseleit (K-H) solution (37 °C) according to the Langendorff technique. After a 30 min equilibration period, vehicle, LPS (1.5 μg/ml) or/and BHT (0.1 μM) were perfused for 90 min. Perfusate TNF-α concentrations, left ventricular+dP/dt and −dP/dt were determined. *n* = 6 for the control or BHT group. *n* = 8 for the LPS or LPS+BHT group. ^#^*P* < 0.05 in comparison to the control group, *P < 0.05 in comparison to the LPS group.

To confirm the role of cardiac endothelial α_2A_-AR in sepsis-induced myocardial dysfunction, we carried out a series of experiments in cultured rat cardiac microvascular endothelial cells (CMECs) and the isolated rat hearts. As shown in Fig. 8A,B, treatment with LPS, an important pathogenicity factor that lead to cardiac depression in experimental sepsis, significantly stimulated TNF-α and VCAM-1 expression in rat CMECs. BHT933, an α_2_-AR agonist, markedly enhanced these effects of LPS on CMECs, which were reversed by pretreatment with α_2A_-AR antagonist, BRL. These findings suggest that α_2A_-AR stimulation promotes LPS-induced cardiac endothelial cell activation. Furthermore, normal rat hearts were perfused in a recirculating mode by the Langendorff procedure, immunofluorescent staining showed that TNF-α- positive vascular endothelial cells in the left ventricle were markedly increased in the hearts perfused with LPS and BHT933 compared with LPS-perfused hearts (Fig. 8C). As shown in Fig. 8D–F, the maximal rate of left ventricular pressure rise and fall (±dP/dt) of control hearts remained relatively stable throughout the perfusion period. After 90 min of recirculating perfusion with LPS, TNF-α concentration in the coronary effluent significantly increased, concomitant with a marked decrease in ±dP/dt compared with control hearts, all of which were further aggravated when both BHT933 and LPS was administered to the recirculating perfusate. Therefore, α_2_-AR stimulation promotes LPS-induced cardiac dysfunction of isolated rat hearts, at least in part, via enhancing cardiac endothelial cell activation.

## Discussion

In the present study, we found that BRL therapy significantly attenuated CLP-induced myocardial dysfunction in rats, as evidenced by increased left ventricular EF, MAP and ± dP/dt as well as reduced cTnI phosphorylation at Ser23/24. To the best of our knowledge, this is the first direct evidence demonstrating the importance of α_2A_-AR inhibition in improving cardiac function during sepsis.

We observed that BRL did not reduce the circulating TNF-α concentration in CLP rats, which is different from earlier reports^10,14^. The different dose and route of BRL administration may contribute to these inconsistent results. However, BRL attenuated CLP-induced myocardial dysfunction. A recent study demonstrated that none of the measured serum cytokines, such as TNF-α, correlated with myocardial dysfunction in septic patients^15^. Accordingly, it is likely that BRL improves CLP-induced cardiac dysfunction through its direct action on the heart. It has been established that myocardial TNF-α and apoptosis contribute to sepsis-induced cardiomyopathy^2,16^, NF-κB and p38 activation mediate LPS-induced TNF-α expression, and JNK phosphorylation involves sepsis-induced apoptosis in cardiomyocytes^16–18^. BRL suppressed myocardial TNF-α production and apoptosis as well as IκBα, p38 and JNK phosphorylation in CLP rats, indicating that α_2A_-AR blockade attenuates septic cardiomyopathy by suppressing myocardial IκBα, p38 and JNK activation, TNF-α production and apoptosis.

It is well documented that α_2A_-AR is present in the cardiac sympathetic nerve presynaptic membrane and blockade of presynaptic α_2A_-AR can augment stimulation-induced NE release^11,19^. Thus, we further detected left ventricle NE concentration in CLP-challenged rats treated with or without BRL. The results showed that CLP challenge for 6 h significantly elevated plasma and cardiac NE contents, which were markedly enhanced by BRL therapy. In order to identify the role of BRL-promoted cardiac release of NE in the protection of BRL against CLP-induced myocardial dysfunction, we exhausted the cardiac NE by reserpine pretreatment. Plasma NE is derived from sympathetic nerve endings, the adrenal gland, the intestine and phagocytes during sepsis. NE recovery in the adrenal gland occurred on the fourth day after RSP injection, and LPS exposure to phagocytes from the mice 4 days after reserpine treatment also caused catecholamine release, but NE recovery was not observed in the heart from the 4^th^ day to the 10^th^ day after RSP injection^13,20,21^. Therefore, we observed that reserpine significantly reduced the cardiac NE level but not the plasma NE level on the 4th day after the last reserpine injection in CLP-challenged rats treated with BRL. Interestingly, reserpine pretreatment did not affect myocardial TNF-α production, IκBα and p38 phosphorylation as well as myocardial dysfunction in CLP-challenged rats, but abolished the inhibitory effects of BRL on myocardial TNF-α production as well as IκBα and p38 phosphorylation, and partially reversed the cardioprotection of BRL in septic rats. These findings indicate that BRL therapy inhibits myocardial TNF-α production as well as IκBα and p38 phosphorylation and improves cardiac dysfunction in septic rats, at least in part, through blocking presynaptic α_2A_-AR thereby promoting cardiac NE release. Because cardiac β_1_-AR activation by NE have been demonstrated to mainly contribute to LPS-induced cardiomyocyte JNK activation and apoptosis during endotoxemia^11,22^, reserpine pretreatment that exhaust cardiac NE significantly reduced myocardial JNK phosphorylation and caspase-3 activation without abrogating the inhibitory effects of BRL on JNK phosphorylation and caspase-3 activation in CLP-challenged rats.

Previous data from our laboratory have also shown that activation of α_1_-AR by NE suppresses LPS-induced cardiomyocyte TNF-α expression and improves cardiac dysfunction during endotoxaemia via suppressing NF-κB and p38 activation^23^, it is likely that increased concentration of cardiac NE by BRL inhibits myocardial TNF-α production and improves cardiac dysfunction via stimulating cardiac α_1_-AR in CLP-challenged rats. To test this hypothesis, we investigated the effect of PRAZ, an α_1_-AR antagonist, on the cardioprotection of BRL in CLP-challenged rats. As we expected, PRAZ administered intraperitoneally just before BRL treatment significantly abrogated the suppressive effects of BRL on myocardial TNF-α production, caspase-3, p38, JNK and IκBα activation as well as cTnI phosphorylation in septic rats, whereas PRAZ administration did not affect these parameters in septic rats, suggesting that BRL therapy promotes cardiac NE release and suppresses myocardial inflammatory cytokine production and apoptosis thereby reducing myocardial dysfunction in sepsis via NE-induced α_1_-AR activation. However, BRL was also demonstrated to activate α_1_-AR^24^. We cannot conclude whether BRL also exerted cardioprotection by directly activating α_1_-AR during sepsis. Furthermore, these protective effects of BRL against sepsis-induced myocardial dysfunction were confirmed by the α_2A_-AR gene deletion using α_2A_-AR KO mice and their wild-tpye controls. These data indicates that α_2A_-AR blockade improves sepsis-induced myocardial dysfunction via promoting cardiac NE release whereby enhancing activation of myocardial α_1_-AR.

However, the deletion of cardiac NE did not completely abolish the inhibitory effect of BRL on CLP-induced myocardial dysfunction. Thus, NE-stimulated α_1_-AR pathway alone cannot fully explain the cardioprotection of BRL therapy during sepsis. It is noteworthy that BRL therapy inhibited CLP-induced myocardial neutrophil infiltration. Moreover, both BRL therapy and α_2A_-AR deficiency reduced MPO content associated with decreased VCAM-1 expression in the left ventricles of CLP-challenged animals, none of which was reversed by reserpine and PRAZ pretreatment. Previous studies have strongly suggested that VCAM-1 mediate LPS-induced myocardial dysfunction independent of neutrophil accumulation in endotoxemic mice^7^. Therefore, it is possible that α_2A_-AR blockade attenuates sepsis-induced myocardial dysfunction through inhibiting cardiac VCAM-1 expression, which was independent of NE-stimulated α_1_-AR pathway. To test this, we further examined the distribution of α_2A_-AR and VCAM-1 in the heart of septic animals. We further found that α_2A_-AR was present in cardiac endothelial cells and that CLP significantly induced VCAM-1 expression in these cardiac endothelial cells, which was markedly suppressed by the pharmacological inhibition or the gene deletion of α_2A_-AR. Previous observations have indicated that endotoxemic myocardial dysfunction may involve cross talk between the coronary endothelial cells and cardiomyocytes, and selective blockade of endothelial-intrinsic NF-κB activation is sufficient to prevent sepsis-induced septic shock^25^. Therefore, α_2A_-AR blockade attenuates sepsis-induced myocardial dysfunction maybe, at least in part, by inhibiting cardiac endothelial activation. In order to verify the contribution of endothelial α_2A_-AR activation to sepsis-induced myocardial dysfunction, we also investigated that the effects of α_2_-AR agonist, BHT933, on endothelial activation and cardiac function in cultured cardiac endothelial cells and isolated rat hearts treated with LPS. We observed that BHT933 treatment enhanced LPS-induced TNF-α mRNA and VCAM-1 protein expression in endothelial cells, both of which were reversed by BRL pretreatment. Moreover, treatment with BHT933 enhances LPS-induced cardiac dysfunction in isolated perfused heart, as evidenced by further decreased ±dP/dt, increased perfusate TNF-α concentration and enhanced endothelial expression of TNF-α. Evidently, BHT933-caused endothelial α_2A_-AR activation promoted LPS-induced cardiac dysfunction. However, the mechanisms by which α_2A_-AR stimulation enhances cardiac endothelial activation during sepsis await investigation.

Recently, β_1_-AR blockade was also found to offer cardioprotection in septic rats^26^. Importantly, β_1_-AR blockade increased the stroke volume and improved 28-day survival in septic patients with heart rate values of more than 95/min when the heart rate was maintained within the range of 80/min to 94/min^27^. Although β-blocker therapy may control the heart rate and reduce the deleterious effects of β-AR stimulation on the heart in septic patients, it potentially negatively affects cardiovascular compensation through negative inotropic and hypotensive effects. BRL was found to have a hypertensive effect and our results demonstrated that α_2A_-AR blockade by BRL not only improved septic cardiomyopathy but also elevated MAP in septic rats. Therefore, α_2A_-AR blockade may be a promising therapeutic approach to septic cardiomyopathy.

## Conclusions

**Figure 9:**
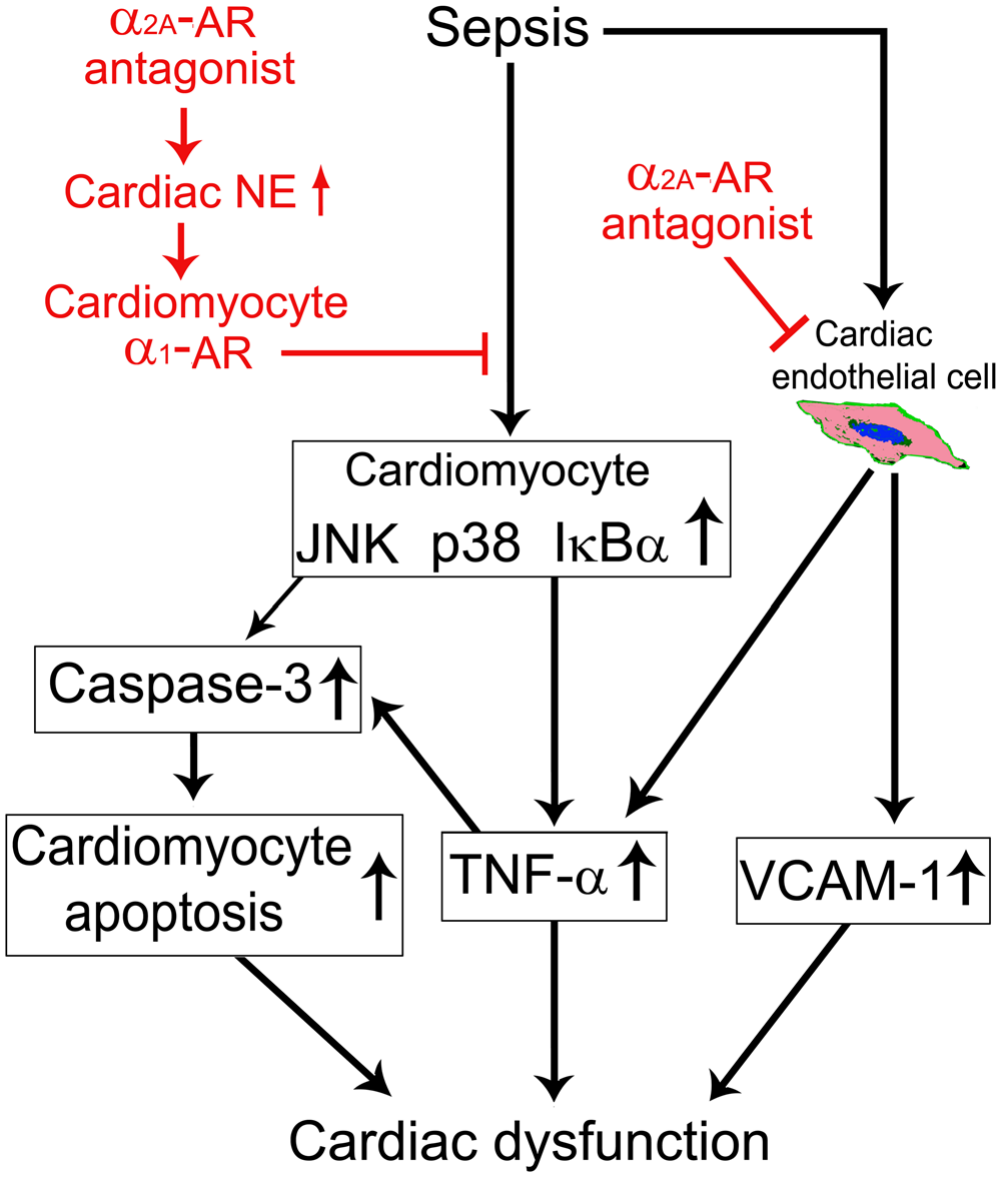
Proposed mechanisms involved in improvement of sepsis-induced cardiac dysfunction by α_2A_-adrenergic blockade. α_2A_-adrenergic receptor (AR) blockade attenuates septic cardiomyopathy by promoting cardiac norepinephrine (NE) release and suppressing cardiac endothelial activation.

This study provides the first direct evidence to demonstrate the causative contribution of cardiac α_2A_-AR activation to septic cardiomyopathy. The inhibition of α_2A_-AR attenuates sepsis-induced cardiomyopathy, which is associated with both increased cardiac NE release that subsequently stimulates myocardial α_1_-AR and suppressed cardiac endothelial activation (Fig. 9).

## Materials and Methods

### Animals and treatments

Specific pathogen-free male adult (8–10-week-old, 250–300 g) Sprague-Dawley rats were purchased from the medical laboratory animal center of Guangdong Province (Guangzhou, China). Eight-to ten-week-old male wild-type (WT, 20–30 g) littermates and α_2A_-AR knockout (α_2A_-AR KO, 20–30 g) mice were generated by *Adra2a*^*tm1Bkk*^ mice (Strain Name: B6.129-*Adra2a*^*tm1Bkk*^/J), which were obtained from Jackson Laboratory (Bar Harbor, ME, USA). The animals were kept under a 12 h light/dark cycle with 60%–80% environment humidity and 24 ± 2 °C room temperature, had free access to food and water, and were allowed to adapt to laboratory conditions for 7 days before experimentation. The experimental protocols were approved by the Experimental Animal Care and Use Committee at Jinan University, which conforms to the Guide for the Care and Use of Laboratory Animals published by the US National Institutes of Health (NIH Publication No. 85–23, revised 1996). All methods were performed in accordance with the relevant guidelines and regulations. All surgeries were performed under anesthesia with isoflurane, and postoperative analgesia was provided with buprenorphine. In BRL experiments, the rats were randomized into sham, CLP, CLP+BRL and sham+BRL (BRL) groups. *n* = 7–10; In RSP experiments, the rats were randomized into sham, CLP, CLP+BRL, RSP+CLP+BRL, RSP+CLP and sham+RSP (RSP) groups. *n* = 7–10; In PRAZ experiments, the rats were divided into sham, CLP, CLP+BRL, CLP+BRL+PRAZ, CLP+PRAZ and PRAZ groups; *n* = 7–10; In α_2A_-AR knockout experiments, the following groups were studied in α_2A_-AR knockout mice and their wild-type (WT) counterparts: sham and CLP. *n* = 9 for each group. In the separate experiments, BRL was injected intraperitoneally 4 h after CLP or sham surgery, and PRAZ (α_1_-AR antagonist, 1.0 mg/kg) was administered intraperitoneally just before BRL (1.5 mg/kg) treatment; The rats first received a subcutaneous injection of RSP (4.5 mg/kg) or normal saline once a day for 2 consecutive days and were then exposed to CLP or sham surgery on the 4th day after the last RSP or normal saline administration. Then, BRL (1.5 mg/kg) or normal saline was injected intraperitoneally 4 h after CLP or sham surgery. BRL, PRAZ and RSP were purchased from Sigma Aldrich (St. Louis, Mo, USA).

### Polymicrobial sepsis model

CLP was performed to induce polymicrobial sepsis in rats and mice as previously described^28^. Briefly, the animals were anesthetized by isoflurane inhalation and an incision was made along the abdominal midline. The cecum was exposed and ligated at 1.0 cm (mice) or 1.5 cm (rats) from the tip of the cecum. A single through-and-through puncture was made in the middle of the ligated cecum using a 14-gauge needle (rats) or a 20-gauge (mice) and a small amount of fecal materials was extruded after removing the needle. Then, the cecum was returned to the abdomen, and the incision was closed in layers. Sham controls received the same surgery without CLP. After surgery, the animals were injected subcutaneously with prewarmed normal saline for fluid resuscitation and buprenorphine for postoperative analgesia.

### Survival study

Four hours after the sham or CLP procedure, the rats were intraperitoneally injected with normal saline (2 mL) or BRL at a dose of 0.75, 1.50 or 3.00 mg/kg (*n* = 10 for each group). Then, the rats were returned to the original cages, allowed free access to food and water, and the survival rate was recorded every 12 hours for up to 10 days. At the end of the survival experiments, the rats were deeply anesthetized with pentobarbital sodium (100 mg/kg) and sacrificed.

### Echocardiography examination

Echocardiographic examination was performed in isofluorane-anesthetized rats at 20 h after sham or CLP surgery using VisualSonics Vevo770 System (VisualSonics Inc, Toronto, ON, Canada) as previously described^29^. Left ventricular (LV) ejection fraction (EF) and end-diastolic volume (LVEDV) were calculated. All echocardiographic measurements were performed by the same qualified technician, and the values were averaged from at least three cardiac cycles.

### Langendorff perfusion of the isolated heart

Left ventricular (LV) functions of the hearts isolated from CLP or sham-operated rats were measured in a Langendorff perfusion apparatus^30^. Briefly, twenty hours after CLP or sham surgery, the rats were heparinized and anesthetized. The hearts were excised, and the aorta was retrograde attached to a Langendorff perfusion system and perfused at 10 ml/min (STH pump controller ML175, ADInstruments, Colorado Springs, CO) with Krebs-Henseleit buffer containing (in mM) 118 NaCl, 4.7 KCl, 25 NaHCO_3_, 1.2 KH_2_PO_4_, 1.2 MgSO_4_, 2.5 CaCl_2_ and 11 glucose (bubbled with 95% O_2_ and 5% CO_2_ gas mixture and maintained at 37 °C). A balloon was inserted into the LV chamber through the mitral valve with an incision in the left atrium and connected to a pressure transducer for the continuous measurement of LV pressure. The LV balloon volume was adjusted to approximately 10 mmHg of the LV end-diastolic pressure for stabilization, and the left ventricular developed pressure and the maximum rates of positive and negative changes in the LV pressure (±dP/dt) were calculated using a data acquisition system.

In separate experiments, Hearts from the normal rats were divided into control (*n* = 6), LPS (*n* = 8), LPS+BHT (*n* = 8) and BHT (*n* = 6) groups, and placed on a Langendorff apparatus and perfused in a recirculating mode with Krebs-Henseleit buffer (total volume, 50 mL). After a 30 min equilibration period, LPS (1.5 μg/mL) or/and BHT933 (a selective α_2_-AR agonist, 0.1 μM, Sigma Aldrich, St. Louis, Mo, USA,) mixed to the Krebs-Henseleit buffer were perfused for 1–2 h. Control hearts were perfused with Krebs-Henseleit buffer. The above physiological parameters of hearts were monitored, and the perfusate and left ventricular myocardial samples were harvested for TNF-α determination and Immunofluorescence staining, respectively.

### Non-invasive measurement of mean arterial pressure

The mean arterial pressure (MAP) was determined in conscious rats using a non-invasive computerized tail cuff system (CODA Non-Invasive Blood Pressure Monitor, Kent Scientific Corporation, Torrington, CT, USA) according to the manufacturer’s instructions. The rats were conditioned to tail cuff instrumentation for 3 days before the experiment to minimize the effects of cuff inflation/deflation stress, and the values were averaged from at least six consecutive cycles.

### Assays for norepinephrine and TNF-α concentration

The concentrations of left ventricular and plasma norepinephrine (NE) and TNF-α were determined using the NE enzyme-linked immunosorbent assay (ELISA) kit (Alpco, Salem, NH, USA) and the TNF-α ELISA kit (R&D Systems, Minneapolis, MN, USA), respectively.

### Western blot analysis

Rat and mouse heart homogenates were harvested in RIPA lysis buffer (Bioteke Co, Beijing, China) containing 1 mM phenylmethylsulfonyl fluoride and then centrifuged at 12,000× *g* for 15 min at 4 °C. Equal amounts of protein were separated by 6%–15% SDS-polyacrylamide gel electrophoresis and transferred on to PVDF membranes (Millipore, Billerica, MA, USA). Following blockage of nonspecific binding sites with 5% nonfat dry milk for 1 h, the membranes were incubated with the appropriate primary antibodies against phosphorylated cardiac troponin I (p-cTnI, Ser23/24), myeloperoxidase (MPO), extracellular signalregulated kinase (ERK) 1/2, phosphorylated ERK1/2 (p-ERK1/2, Thr202/Tyr204), c-jun NH2-terminal kinase (JNK)1/2, phosphorylated JNK1/2 (p-JNK1/2, Thr183/Tyr185), p38, phosphorylated p38 (p-p38, Thr180/Tyr182), IκBα, phosphorylated IκBα (p-IκBα, Ser32), cleaved-caspase-3, caspase-3, glyceraldehyde-3-phosphate dehydrogenase (GAPDH, Cell Signaling Technology, MA, USA), vascular cell adhesion molecule-1 (VCAM-1) or cTnI (Abcam plc, Cambridge, UK) overnight at 4 °C, followed by incubation with a horseradish peroxidase-conjugated IgG secondary antibody. The immunoreactive bands were visualized with an enhanced chemiluminescence reagent (Millipore, Billerica, MA, USA), and their intensities were determined by densitometry.

### Immunofluorescence staining

The animals were killed at the indicated time-points after CLP. The hearts were harvested, fixed in 4% paraformaldehyde, infiltrated with Tissue Tek OCT compound and rapidly frozen before sectioning. The sections were blocked in PBS with 5% donkey serum and incubated with different dilutions of antibodies against TNF-α (Santa Cruz, CA, USA), Ly6G (a positive marker for neutrophils), α_2A_-AR, CD31 (a positive marker for endothelial cells), and/or VCAM-1 (Abcam plc, Cambridge, UK) with different dilution at 4 °C overnight. Then, some sections were incubated with a 1:100 dilution of antibody against cTnI (Abcam plc, Cambridge, UK) at 4 °C overnight. After 3 washes with PBS, the sections were incubated with a 1:1000 dilution of secondary antibodies conjugated with Alexa Fluor^®^ dyes (Invitrogen, Carlsbad, Calif, USA) for 1 h and observed using a laser-scanning confocal microscope (LSM510META; Zeiss, Oberkochen, Germany).

### Terminal deoxynucleotidyl transferase-mediated dUTP nick-end-labeling assay

Cardiomyocyte apoptosis was determined using a terminal deoxynucleotidyl transferase-mediated dUTP nick-end-labeling (TUNEL) assay using an *in situ* cell death detection kit (Roche Applied Science, Indianapolis, IN, USA) according to the manufacturer’s instructions. Briefly, tissue sections (5 μm) from frozen cardiac tissues were permeabilized in 0.1% Triton X-100 in 0.1% sodium citrate for 2 min at 4 °C. Triple staining with TUNEL, anti-cTnI and DAPI was performed. Then, the sections were observed by using a laser-scanning confocal microscope.

### Culture and treatment of cardiac microvascular endothelial cells

Adult male Sprague-Dawley rats (4-week-old) were anesthetized and the hearts were rapidly excised. Left ventricles were immersed in 70% ethanol for 30 s to devitalize endocardial endothelial cells and epicardial mesothelial cells, and then extensively washed with calcium-free Krebs-Henseleit bicarbonate buffer. Cardiac microvascular endothelial cells were enzymatically isolated from left ventricular tissue using 0.2% collagenase type II (Sigma-Aldrich, St.Louis, MO, USA) and 0.10% trypsin (Thermo Fisher Scientific, Waltham, MA, USA). Dissociated cells were filtered and suspended in Medium 131 containing microvascular growth supplements (Thermo Fisher Scientific, Waltham, MA, USA) and antibiotics. Cultures were maintained at 37 °C in a humidified 95% air and 5% CO_2_ atmosphere, and microvascular endothelial cells in the third and fourth passage were used. The cardiac microvascular endothelial cells were identified by immunostaining for cellular markers, including Factor VIII, CD31, α-smooth muscle actin (α-SMA), vimentin and desmin.

Purified cardiac microvascular endothelial cells were preincubated with BRL (0.2 μM) or vehicle for 30 min and with vehicle or BHT (0.1 μM) for another 30 min, and then treated with or without LPS (1 µg/ml) for 2 or 12 h, the expression of TNF-α mRNA (2 h after LPS treatment) and VCAM-1 protein (12 h after LPS treatment) were determined

### Real-time reverse transcription-polymerase chain reaction (RT-PCR) assay

Gene expression levels of TNF-α in cardiac microvascular endothelial cells were quantified using RT-PCR assay. In brief, total RNA was isolated using the Trizol reagent ((Invitrogen, Carlsbad, CA, USA) and reverse transcribed using a PrimeScript® RT reagent kit. RT-PCR were performed with the SYBR® PrimeScript^TM^ RT-PCR Kit II (TaKa-Ra, Kyoto, Japan), in a LC480 real-time PCR system (Roche, Basel, Switzerland). The sequence of each primer is shown as follows: TNF-α (Forward 5′-CGTCAGCCGATTTGCCATTT-3′, reverse 5′-TCCCTCAGGGGTGTCCTTAG-3′), GAPDH (forward 5′-AGGACCAGGTTG TCTCCTGT-3′, reverse 5′-CCATGT AGGCCATGAGGTCC-3′). The expression of each gene was normalized to that of GAPDH mRNA and results are expressed as the fold increase over controls.

### Statistical analysis

The data were expressed as the mean ± S.E.M. and analyzed using statistical software SPSS 13.0 (SPSS Inc., Chicago, IL, USA). The statistical significance of the difference between the two groups was measured by a two-tailed independent Student’s *t*-test or ANOVA with Bonferroni post hoc analysis, as appropriate. Survival rates were compared using the Kaplan-Meier survival analysis with log-rank tests. Statistical significance was accepted at *P* < 0.05.
